# Gap functions for quasi-variational inequalities via duality

**DOI:** 10.1186/s13660-017-1603-9

**Published:** 2018-01-10

**Authors:** L Altangerel

**Affiliations:** German-Mongolian Institute for Resources and Technology, Nalaikh, Mongolia

## Abstract

This paper deals with an application of duality theory in optimization to the construction of gap functions for quasi-variational inequalities. The same approach was investigated for variational inequalities and equilibrium problems in (Pac. J. Optim. 2(3): 667-678, 2006; Asia-Pac. J. Oper. Res. 24(3): 353-371, 2007), and the study shows that we can obtain some previous results for variational inequalities as special cases. Moreover, some applications dealing with the generalized Nash equilibrium problems and mixed variational inequalities are presented.

## Introduction

The quasi-variational inequality which is a generalization of the variational inequality problem was introduced first by Bensoussan et al. in [3] in the context of impulse control problems. The quasi-variational inequalities have many applications in economics, game theory, optimization and other applied sciences. It is well known that the generalized Nash equilibrium problem can be reduced to the quasi-variational inequality problem (see [4]).

A gap function approach is one of the main tools for solving variational inequalities. Different approaches on gap functions for quasi-variational inequalities have been investigated by various authors [5–9]. On the other hand, by using different dual problems in convex optimization (see [10, 11]), gap functions for variational inequalities and equilibrium problems have been investigated in [1, 2]. Specially, in [2], based on the conjugate duality for optimization problem, some gap functions for mixed variational inequalities, also dual gap functions for the variational inequality and the relation between these functions have been investigated. However, it still remains an open question how the same approach can be extended to quasi-variational inequalities. This paper aims to answer this question by applying duality results from [12] which deals with minimization of a convex function over the solution set of a range inclusion problem determined by a set-valued mapping.

The paper is organized as follows. Section 2 deals with some preliminary results from [12]. In Section 3 we consider the duality based approach on gap functions for quasi-variational inequalities. Section 4 is devoted to the investigation of gap functions for mixed quasi-variational inequalities. Finally, some applications dealing with mixed variational inequalities and the generalized Nash equilibrium problems are presented in Section 5.

## Preliminaries

We consider the following optimization problem:

$$(P)\quad\inf_{0\in F(x)} f(x), $$

where $f:\mathbb{R}^{n} \rightarrow\overline{\mathbb{R}}$ is a given function and $F:\mathbb{R}^{n}\rightrightarrows\mathbb{R}^{n} $ is a set-valued mapping such that $\operatorname{dom}f\cap F^{-1}(0)\neq \varnothing$.

Then the corresponding dual problem becomes

$$(D)\quad\sup_{p\in\mathbb{R}^{n}}\inf_{x\in\mathbb{R}^{n}} \bigl[f(x)+s_{F}(x,p)\bigr], $$

where $s_{F}:\mathbb{R}^{n}\times\mathbb{R}^{n}\rightarrow\overline {\mathbb{R}}$ is the lower support function associated with *F* defined by

$$s_{F}(x,p)=\inf_{y\in F(x)} \langle p, y \rangle, $$

and $\langle\cdot,\cdot\rangle$ is the Euclidean inner product.

### Proposition 1

([12])

*Let* $f:\mathbb{R}^{n}\rightarrow\overline{\mathbb{R}}$ *be a proper and convex function and* $F:\mathbb{R}^{n}\rightrightarrows\mathbb {R}^{n}$ *be a convex set*-*valued mapping*. *If the constraint qualification*

$$(\mathit{CQ})\quad\exists\bar{x}\in \operatorname{ri}(\operatorname{dom}f)\cap \operatorname{ri}(\operatorname{dom}F)\quad \textit{and}\quad 0\in \operatorname{ri}\bigl(F( \bar{x})\bigr) $$

*is fulfilled*, *then for* $(P)$ *and* $(D)$ *strong duality holds*, *i*.*e*., $\exists\bar{p}\in\mathbb{R}^{n}$ *such that*

$$\begin{aligned} \inf_{0\in F(x)} f(x) =&\sup_{p\in\mathbb{R}^{n}}\inf _{x\in\mathbb {R}^{n}} \bigl[f(x)+s_{F}(x,p)\bigr] \\ =&\inf_{x\in\mathbb{R}^{n}}\bigl[f(x)+s_{F}(x,\bar{p})\bigr], \end{aligned}$$

*where* dom*h* *is the effective domain of a given function* $h:\mathbb{R}^{n}\rightarrow\overline{\mathbb{R}}$ *and* $\operatorname{ri}(C)$ *is the relative interior of a given set* $C\subseteq\mathbb{R}^{n}$.

## Gap function for quasi-variational inequalities

Let $T:\mathbb{R}^{n}\rightarrow\mathbb{R}^{n}$ be a continuous vector-valued function and $K:\mathbb{R}^{n}\rightrightarrows\mathbb {R}^{n}$ be a set-valued mapping such that $K(x)$ is nonempty, closed and convex for each $x\in\mathbb{R}^{n}$. Then the quasi-variational inequality problem consists in finding a vector $x\in K(x)$ such that

$$(\mathit{QVI})\quad\bigl\langle T(x),y-x\bigr\rangle \geq0,\quad\forall y\in K(x). $$

For a fixed $x\in\mathbb{R}^{n}$, $(\mathit{QVI})$ can be rewritten as an optimization problem

$$\bigl(P^{\mathit{QVI}};x\bigr)\quad\inf_{y\in K(x)} \bigl\langle T(x),y-x\bigr\rangle . $$

Let us define a function $\gamma^{\mathit{QVI}}(x):\mathbb{R}^{n}\rightarrow \overline{\mathbb{R}}$ for $x\in\mathbb{R}^{n}$ (cf. [2])

$$\gamma^{\mathit{QVI}}(x):=-v\bigl(D^{\mathit{QVI}};x\bigr), $$

where $v(D^{\mathit{QVI}};x)$ denotes the optimal objective value of the dual problem for $(P^{\mathit{QVI}};x)$. Since $(P^{\mathit{QVI}};x)$ can be reformulated as

$$\inf_{0\in K(x)-y}\bigl\langle T(x),y-x\bigr\rangle , $$

the dual problem for $(P^{\mathit{QVI}};x)$ turns out to be

$$\begin{aligned} \bigl(D^{\mathit{QVI}};x\bigr)\quad &\sup_{p\in\mathbb{R}^{n}}\inf _{y\in\mathbb {R}^{n}}\bigl[\bigl\langle T(x),y-x\bigr\rangle +s_{K(x)-\mathit{id}}(y,p) \bigr] \\ &\quad =\sup_{p\in\mathbb{R}^{n}}\inf_{y\in\mathbb{R}^{n}}\bigl[\bigl\langle T(x),y\bigr\rangle +s_{K(x)-\mathit{id}}(y,p) \bigr]-\bigl\langle T(x),x\bigr\rangle . \end{aligned}$$

Consequently, we have

$$\gamma^{\mathit{QVI}}(x)=\bigl\langle T(x),x\bigr\rangle -\sup _{p\in\mathbb{R}^{n}}\inf_{y\in\mathbb{R}^{n}}\bigl[\bigl\langle T(x),y\bigr\rangle +s_{K(x)-\mathit{id}}(y,p) \bigr]. $$

Let us recall now the definition of a gap function for quasi-variational inequalities and give an auxiliary result.

### Definition 1

A function $\gamma: \mathbb{R}^{n} \rightarrow\overline{\mathbb{R}}$ is said to be a gap function for the problem $(\mathit{QVI})$ if it satisfies the following properties:

(i) $\gamma(y) \geq0$, $\forall y \in K(x)$;
(ii) $\gamma(x) = 0$ if and only if x solves the problem $(\mathit{QVI})$.

### Lemma 1

*Let* $K:\mathbb{R}^{n}\rightrightarrows\mathbb{R}^{n}$ *be a set*-*valued mapping and* $p\in\mathbb{R}^{n}$ *be fixed*. *Then*, *for any* $x\in\mathbb {R}^{n}$, *it holds*

$$s_{K(x)-\mathit{id}}(y,p)=s_{K}(x,p)-\langle p,y\rangle. $$

### Proof

Let $x\in\mathbb{R}^{n}$ and $p\in\mathbb{R}^{n}$ be fixed. Then, by definition, we have

$$\begin{aligned}& s_{K(x)-\mathit{id}}(y,p)=\inf_{z\in K(x)-y}\langle p,z\rangle \\& (z+y:=t) =\inf_{t\in K(x)}\langle p,t-y\rangle= \inf _{t\in K(x)}\langle p,t\rangle-\langle p,y\rangle =s_{K}(x,p)-\langle p,y\rangle. \end{aligned}$$

 □

### Proposition 2

*For the problem* $(\mathit{QVI})$, *we have*

$$\gamma^{\mathit{QVI}}(x)= \textstyle\begin{cases} -\inf_{y\in F(x)}\langle T(x), y-x\rangle,&p=T(x),\\ +\infty,&\textit{otherwise}. \end{cases} $$

### Proof

By using Lemma 1, one obtains that

$$\begin{aligned} \gamma^{\mathit{QVI}}(x) =&\bigl\langle T(x),x\bigr\rangle -\sup _{p\in\mathbb {R}^{n}}\inf_{y\in\mathbb{R}^{n}}\bigl[\bigl\langle T(x),y\bigr\rangle +s_{K(x)-\mathit{id}}(y,p) \bigr] \\ =&\bigl\langle T(x),x\bigr\rangle -\sup_{p\in\mathbb{R}^{n}} \Bigl[s_{K}(x,p)+\inf_{y\in\mathbb{R}^{n}}\bigl\langle T(x)-p,y\bigr\rangle \Bigr]. \end{aligned}$$

From

$$\inf_{y\in\mathbb{R}^{n}} \bigl\langle T(x)-p,y\bigr\rangle = \textstyle\begin{cases} 0,&T(x)-p=0,\\ -\infty,&\text{otherwise}, \end{cases} $$

it follows that

$$\begin{aligned} \gamma^{\mathit{QVI}}(x) =& \textstyle\begin{cases} \langle T(x),x\rangle-\sup_{p\in\mathbb{R}^{n}}s_{K}(x,T(x)),&p=T(x),\\ +\infty,&\text{otherwise} \end{cases}\displaystyle \\ =& \textstyle\begin{cases} \langle T(x),x\rangle-\inf_{y\in F(x)}\langle T(x), y\rangle ,&p=T(x),\\ +\infty,&\text{otherwise} \end{cases}\displaystyle \\ =& \textstyle\begin{cases} -\inf_{y\in F(x)}\langle T(x), y-x\rangle,&p=T(x),\\ +\infty,&\text{otherwise}. \end{cases}\displaystyle \end{aligned}$$

 □

### Remark 1

A gap function $\gamma(x)=-\inf_{y\in F(x)}\langle T(x),y-x\rangle$ was investigated in [7] (see also [6]).

## Gap functions for mixed quasi-variational inequalities

Let $T:\mathbb{R}^{n}\rightarrow\mathbb{R}^{n}$ be a continuous vector-valued function and $K:\mathbb{R}^{n}\rightrightarrows\mathbb {R}^{n}$ be a set-valued mapping such that $K(x)$ is nonempty, closed and convex for each $x\in\mathbb{R}^{n}$. Let $\varphi:\mathbb {R}^{n}\rightarrow\overline{\mathbb{R}}$ be a given function. Then the mixed quasi-variational inequality problem consists in finding a vector $x\in K(x)$ such that (cf. [13])

$$(\mathit{MQVI})\quad\bigl\langle T(x),y-x\bigr\rangle +\varphi(y)-\varphi(x)\geq 0,\quad \forall y\in K(x). $$

Rewriting $(\mathit{MQVI})$ as an optimization problem

$$\bigl(P^{\mathit{MQVI}};x\bigr)\quad\inf_{y\in K(x)} \bigl[\bigl\langle T(x),y-x\bigr\rangle +\varphi (y)-\varphi(x)\bigr] $$

and repeating the same techniques in Section 3, we can define the following function:

$$\gamma^{\mathit{MQVI}}(x)=-\sup_{p\in\mathbb{R}^{n}}\inf_{y\in\mathbb {R}^{n}} \bigl[\bigl\langle T(x),y-x\bigr\rangle +\varphi(y)-\varphi (x)+s_{K(x)-\mathit{id}}(y,p) \bigr]. $$

It can be rewritten as

$$\begin{aligned} \gamma^{\mathit{MQVI}}(x) =&\bigl\langle T(x),x\bigr\rangle +\varphi(x)-\sup_{p\in\mathbb{R}^{n}}\inf_{y\in\mathbb{R}^{n}}\bigl[\bigl\langle T(x),y\bigr\rangle +\varphi(y)+s_{K}(x,p)-\langle p,y\rangle\bigr] \\ =&\bigl\langle T(x),x\bigr\rangle +\varphi(x)+\inf_{p\in\mathbb{R}^{n}}[-s_{K}(x,p)-\inf_{y\in\mathbb {R}^{n}} \bigl[\bigl\langle T(x)-p,y\bigr\rangle -\varphi(y)\bigr] \\ =&\bigl\langle T(x),x\bigr\rangle +\varphi(x)+\inf_{p\in\mathbb {R}^{n}} \bigl[-s_{K}(x,p)+\varphi^{*}\bigl(p-T(x)\bigr)\bigr], \end{aligned}$$

where $h^{*}:\mathbb{R}^{n}\rightarrow\overline{\mathbb{R}}$ is the conjugate function of a given function $h:\mathbb{R}^{n}\rightarrow \overline{\mathbb{R}}$ defined by $h^{*}(p)=\sup_{x\in\mathbb{R}^{n}}[\langle p,x\rangle-h(x)]$.

### Theorem 1

*Let* $K:\mathbb{R}^{n}\rightrightarrows\mathbb{R}^{n}$ *be a set*-*valued mapping such that* $K(x)$ *is nonempty*, *closed and convex for each* $x\in \mathbb{R}^{n}$ *and* *φ* *be convex*. *If*, *for each* $x\in\mathbb{R}^{n}$, *the constraint qualification*

$$(\mathit{CQ};x)\quad\exists\bar{x}\in \operatorname{ri}(\operatorname{dom}\varphi)\quad \textit{and}\quad0\in \operatorname{ri}\bigl(K(x)-\bar{x}\bigr) $$

*is fulfilled*, *then* $\gamma^{\mathit{MQVI}}$ *is a gap function for* $(\mathit{MQVI})$.

### Proof

(i) Let $x\in\mathbb{R}^{n}$ be fixed. By weak duality, one gets
  $$v\bigl(D^{\mathit{MQVI}},x\bigr)\leq v\bigl(P^{\mathit{MQVI}},x\bigr)\leq0, $$
  where $(D^{\mathit{MQVI}};x)$ is a dual problem for $(P^{\mathit{MQVI}};x)$. Consequently, we have $\gamma^{\mathit{MQVI}}(x)=-v(D^{\mathit{MQVI}},x)\geq0$.
(ii) If $\gamma^{\mathit{MQVI}}(x)=0$, then
  $$0=v\bigl(D^{\mathit{MQVI}},x\bigr)\leq v\bigl(P^{\mathit{MQVI}},x\bigr)\leq0. $$
  In other words, $v(P^{\mathit{MQVI}},x)=0$, which means that *x* is a solution of $(\mathit{MQVI})$. Conversely, if *x* is a solution to the problem $(\mathit{MQVI})$, then $v(P^{\mathit{MQVI}},x)=0$. By Proposition 1, we obtain that
  $$\gamma^{\mathit{MQVI}}(x)=-v\bigl(D^{\mathit{MQVI}},x\bigr)=-v\bigl(P^{\mathit{MQVI}},x \bigr)=0. $$

 □

### Remark 2

Let us assume that $T:\mathbb{R}^{n}\rightarrow\mathbb{R}^{n}$ is affine and $K:\mathbb{R}\rightrightarrows\mathbb{R}^{n}$ is a set-valued mapping. It is easy to check that $s_{K}(x,p)$ is concave with respect to *p*. By assumption, $\varphi^{*}(p-T(x))$ is convex, and therefore, the last term in $\gamma^{\mathit{MQVI}}$ is a convex optimization problem for fixed $x\in\mathbb{R}^{n}$:

$$\inf_{p\in\mathbb{R}^{n}}\bigl[-s_{K}(x,p)+\varphi^{*}\bigl(p-T(x) \bigr)\bigr]. $$

### Remark 3

Let $K(x)\equiv K$. Then we have the mixed variational inequality which consists in finding a vector $x\in K$ such that

$$(MVI)\quad\bigl\langle T(x),y-x\bigr\rangle +\varphi(y)-\varphi(x)\geq0, \quad \forall y\in K. $$

In this case, we have $s_{K}(x,p)=-\delta_{K}^{*} (-p)$, where $\delta_{K}:\mathbb{R}^{n}\rightarrow\overline{\mathbb{R}}$

$$\delta_{K}(y)= \textstyle\begin{cases} 0,&\mbox{if } y\in K,\\ +\infty,&\text{otherwise}, \end{cases} $$

denotes the indicator function of the set *K*. Consequently, one obtains

$$\gamma^{MVI}(x)=\bigl\langle T(x),x\bigr\rangle +\varphi(x)+\inf _{p\in\mathbb {R}^{n}}\bigl[\delta_{K}(-p)+\varphi^{*}\bigl(p-T(x) \bigr)\bigr], $$

which is nothing else than the one investigated in [2].

## Special cases

### The generalized Nash equilibrium problems

The generalized Nash equilibrium problem (GNEP for short) is an extension of the classical Nash equilibrium problem, in which each player’s strategy set depends on the rival player’s strategies. We refer to [14] for the excellent comprehensive surveys on theories and algorithms for GNEP. We consider *N* player’s game. Each player *k* controls his decision variable $x^{k}\in\mathbb{R}^{n_{k}}$, $n_{k}\in\mathbb{N}$, such that the vector $x=(x^{1},\dots,x^{N})\in \mathbb{R}^{n}$ with $n=n_{1}+n_{2}+\cdots +n_{N}$ describes the decision vector of all players. We oft use the notation $x=(x^{k},x^{-k})$, where $x^{-k}=(x^{1},x^{2},\ldots,x^{k-1},x^{k+1},\ldots,x^{N})$. Furthermore, each player *k* has a cost function $\theta_{k}: \mathbb{R}^{n}\rightarrow \mathbb{R}$ and a strategy set $X_{k}(x^{-k})\subseteq\mathbb {R}^{n_{k}}$ defined by the set-valued mapping $X_{k}:\mathbb {R}^{n-n_{k}}\rightrightarrows\mathbb{R}^{n_{k}}$. Let

$$\Omega(x):=X_{1}\bigl(x^{-1}\bigr)\times\cdots\times X_{N}\bigl(x^{-N}\bigr). $$

Then $(\mathit{GNEP})$ consists in finding a vector $\bar{x}=(\bar{x}^{1},\ldots,\bar{x}^{N})$ such that, for each $k=\overline{1,N}$, the vector $\bar{x}^{k}$ solves

$$P_{k}\bigl(\bar{x}^{-k}\bigr)\quad \inf_{x^{k}\in X_{k}(\bar{x}^{-k})} \theta\bigl(x^{k},\bar{x}^{-k}\bigr). $$

We assume that $X_{k}\subseteq\mathbb{R}^{n_{k}}$, $k=1,\ldots,N$, are nonempty, closed and convex sets and, for each fixed $x^{-k}\in \mathbb{R}^{n-n_{k}}$, the functions $\theta_{k}(\cdot, x^{-k})$ are convex and differentiable.

Let us define the vector-valued function $F:\mathbb{R}^{n}\rightarrow \mathbb{R}^{n}$ by

$$F(x)= \bigl(\nabla_{x^{k}} \theta_{k}\bigl(x^{k},x^{-k} \bigr) \bigr)_{k=1}^{N}. $$

Then it is well known that (see [4]) $(\mathit{GNEP})$ is reduced to the problem of finding a vector $\bar{x} \in\Omega(\bar{x})$ such that

1 $$ \bigl\langle F(\bar{x}), y-\bar{x} \bigr\rangle \geq0,\quad \forall y \in\Omega (\bar{x}). $$

#### Proposition 3

(cf. [12])

*Assume that* $\exists\bar{x}\in K(\bar{x})$ *and* $\bar{p}\in\mathbb{R}^{n}$ *satisfying the following conditions*:

(i) $\sum_{k=1}^{N} \langle\nabla_{x^{k}}\theta_{k}(\bar{x}^{k},\bar{x}^{-k}), \bar{x}^{k}\rangle=\inf_{y\in\mathbb{R}^{n}} [\sum_{k=1}^{N} \langle\nabla_{x^{k}}\theta_{k}(\bar{x}^{k},\bar{x}^{-k}) ,y^{k}\rangle+s_{\Omega(\bar{x})-\mathit{id}}(y,\bar{p}) ]$;
(ii) $s_{\Omega(\bar{x})-\mathit{id}}(\bar{x},\bar{p})=0$.

*Then* *x̄* *is a solution of* $(\mathit{GNEP})$.

#### Proof

Let $x\in K(x)$, $p\in\mathbb{R}^{n}$ be fixed and conditions (i)-(ii) in Proposition 2 be fulfilled. Then, according to Lemma 1, condition (i) can be rewritten as

$$\begin{aligned}& \begin{gathered} \sum_{k=1}^{N} \bigl\langle \nabla_{x^{k}}\theta_{k}\bigl(x^{k},x^{-k} \bigr), x^{k}\bigr\rangle +s_{\Omega(x)-\mathit{id}}(x,p) \\ \quad =\inf_{y\in\mathbb{R}^{n}} \Biggl[\sum_{k=1}^{N} \bigl\langle \nabla_{x^{k}}\theta_{k}\bigl(x^{k},x^{-k} \bigr) ,y^{k}\bigr\rangle +s_{\Omega(x)-\mathit{id}}(y,p) \Biggr] \end{gathered} \\& \quad \Leftrightarrow\quad \sum_{k=1}^{N} \bigl\langle \nabla_{x^{k}}\theta _{k}\bigl(x^{k},x^{-k} \bigr), x^{k}\bigr\rangle +s_{\Omega}(x,p)-\langle p,x\rangle \\& \qquad \hphantom{\Leftrightarrow\quad }=\inf_{y\in\mathbb{R}^{n}} \Biggl[\sum_{k=1}^{N} \bigl\langle \nabla_{x^{k}}\theta_{k}\bigl(x^{k},x^{-k} \bigr) ,y^{k}\bigr\rangle +s_{\Omega}(x,p)-\langle p,y\rangle \Biggr] \\& \quad \Leftrightarrow\quad \sum_{k=1}^{N} \bigl\langle \nabla_{x^{k}}\theta _{k}\bigl(x^{k},x^{-k} \bigr)-p^{k}, x^{k}\bigr\rangle =\sum _{k=1}^{N} \inf_{y^{k}\in\mathbb{R}^{n_{k}}} \bigl\langle \nabla_{x^{k}}\theta_{k}\bigl(x^{k},x^{-k} \bigr)-p^{k} ,y^{k}\bigr\rangle \\& \quad \Leftrightarrow\quad \bigl\langle \nabla_{x^{k}}\theta_{k} \bigl(x^{k},x^{-k}\bigr)-p^{k}, x^{k}\bigr\rangle =\inf_{y^{k}\in\mathbb{R}^{n_{k}}} \bigl\langle \nabla_{x^{k}} \theta_{k}\bigl(x^{k},x^{-k}\bigr)-p^{k} ,y^{k}\bigr\rangle ,\quad k=\overline{1,N}. \end{aligned}$$

From

$$\inf_{y^{k}\in\mathbb{R}^{n_{k}}} \bigl\langle \nabla_{x^{k}} \theta_{k}\bigl(x^{k},x^{-k}\bigr)-p^{k} ,y^{k}\bigr\rangle = \textstyle\begin{cases} 0,&\nabla_{x^{k}}\theta_{k}(x^{k},x^{-k})-p^{k}=0,\\ -\infty,&\text{otherwise}, \end{cases} $$

it follows that

$$p^{k}=\nabla_{x^{k}}\theta_{k}\bigl(x^{k},x^{-k} \bigr), \quad k=\overline{1,N}, $$

or, equivalently,

2 $$ p=F(x). $$

Setting (2) in condition (ii), we obtain that

$$\inf_{z\in\Omega(x)-x}\bigl\langle F(x),z\bigr\rangle =0, $$

which is nothing else than (1). □

### Linear mixed variational inequality

Recently, the following type of mixed variational inequality has been investigated intensively with particular interest in studying electrical circuits involving transistors (see [15] and [16]): to find a vector $x\in\mathbb{R}^{n}$ such that

$$(\mathit{LMVI})\quad\langle Mx+q,y-x\rangle+\varphi(y)-\varphi(x)\geq0, \quad\forall y\in \mathbb{R}^{n}, $$

where $M\in\mathbb{R}^{m\times n}$ is a real *P*-matrix and $q\in \mathbb{R}^{n}$. Since

$$\delta_{\mathbb{R}^{n}}(-p)=\sup_{y\in\mathbb{R}^{n}} \langle -p,y\rangle= \textstyle\begin{cases} 0,&p=0,\\ +\infty,&\text{otherwise}, \end{cases} $$

we get

$$\gamma^{\mathit{LMVI}}(x)=\langle Mx+q,x\rangle+\varphi(x)+\varphi^{*}(-Mx-q)]. $$

By definition of a gap function,

$$\gamma^{\mathit{LMVI}}(x)=0\quad\Leftrightarrow\quad Mx+q\in\partial\varphi (x) \quad \Leftrightarrow\quad0\in-Mx-q-\partial\varphi(x). $$
